# Efficacy of Dietary Lipid Control in Healing High-Fat and High-Cholesterol Diet-Induced Fibrotic Steatohepatitis in Rats

**DOI:** 10.1371/journal.pone.0145939

**Published:** 2016-01-04

**Authors:** Hazuki Tamada, Hisao Naito, Kazuya Kitamori, Yumi Hayashi, Nozomi Yamagishi, Masashi Kato, Tamie Nakajima

**Affiliations:** 1 Department of Human Life and Environment, Kinjo Gakuin University, Nagoya, Japan; 2 Department of Public Health, Fujita Health University School of Medicine, Nagoya, Japan; 3 Department of Pathophysiological Laboratory Sciences, Nagoya University Graduate School of Medicine, Nagoya, Japan; 4 Department of Occupational and Environmental Health, Nagoya University Graduate School of Medicine, Nagoya, Japan; 5 College of Life and Health Sciences, Chubu University, Kasugai, Japan; University of Catania, ITALY

## Abstract

Nonalcoholic steatohepatitis is related to lifestyle, particularly to dietary habits. We developed diet-induced fibrotic steatohepatitis model stroke-prone spontaneously hypertensive 5/Dmcr (SHRSP5/Dmcr) rats showing steatosis, hepatic inflammation, and severe fibrosis induced by high-fat and -cholesterol (HFC) diet feeding. We aimed to clarify the efficacy of dietary intervention on the disease before and after the appearance of fibrosis. Male SHRSP5/Dmcr rats were divided into 9 groups; of these, 6 groups were fed control or HFC diet for several weeks and the remaining 3 groups represented the dietary intervention groups, which were fed the control diet after HFC diet feeding for 2 (before the appearance of fibrosis) or 8 (after the appearance of fibrosis) weeks. Dietary intervention before the appearance of fibrosis significantly improved the steatosis and reset the increased serum aspartate aminotransferase (AST), alanine aminotransferase (ALT), and serum total cholesterol (TC) levels. However, dietary intervention after the appearance of fibrosis was unable to reset the levels of hepatic TC, serum ALT, and fibrogenesis-related markers and had only a minor influence on hepatic fibrosis, although it reset the increased expression of transforming growth factor (TGF)-β1 and α-smooth muscle actin (SMA). It was noted that dietary intervention improved the increased AST levels; however, aggregated CD68-positive cells were still observed around the fibrosis area, which may be related to the findings of inflammatory cytokine mRNAs. Taken together, dietary intervention for fibrotic steatohepatitis improved steatosis, although it could not completely improve fibrosis.

## Introduction

Nonalcoholic steatohepatitis (NASH) is a severe form of nonalcoholic fatty liver disease (NAFLD), which includes a wide spectrum of conditions from simple steatosis to hepatic fibrosis [1,2]. Some forms of the disease can progress into cirrhosis and hepatocellular carcinoma [3]. The increased prevalence of NAFLD/NASH is a major issue in Japan as well as other countries [4,5]. NAFLD/NASH is closely related to lifestyle, particularly to dietary habits, obesity, and type 2 diabetes, and is considered to be a hepatic manifestation of metabolic syndrome [6,7,8]. However, even patients without obesity and type 2 diabetes sometimes suffer from this disease [9]. Therefore, we should investigate the pathogenesis and progression in patients with/without these risks.

To date, several animal models for NAFLD/NASH have been reported, including dietary [10,11,12,13,14,15,16], chemical [17], and genetic models [18,19,20]. However, these models do not always reflect the relationship between lifestyle and NAFLD/NASH because many of them use a methionine- and choline-deficient diet, which is not an actual diet pattern, or chemical compounds such as dimethylnitrosamine. We have established a new animal model showing fibrotic steatohepatitis by feeding only a high-fat and -cholesterol (HFC) diet to stroke-prone spontaneously hypertensive 5/Dmcr (SHRSP5/Dmcr) rats [21,22]. This strain did not have obesity or diabetes, but had HFC diet-induced steatosis, lobular inflammation, and hepatic fibrosis in a duration dependent manner. Therefore, it is a relevant experimental model for NAFLD/NASH, and it is well-matched to the lifestyle of the patients.

Although there have been few studies quantitatively assessing the relationship between dietary efficacy and the mechanisms of NASH, the first choice of treatment is dietary intervention because NASH is a lifestyle-related disease. A combination of dietary intervention with exercise therapy for NASH patients has been reported to improve the biochemical and histological status [23,24] and is superior to exercise therapy alone [25]. These results suggest that the importance of dietary intervention goes beyond exercise therapy. However, excessive energy restriction, including fasting deteriorated hepatic fibrosis, has been noted [26,27]; therefore, an appropriate balanced energy intake and body weight control for patients with NAFLD/NASH is recommended to prevent disease progression [28].

From this point of view, our study aimed to evaluate the efficacy of dietary intervention, particularly that of dietary lipid control, with enough energy for improvement of HFC diet-induced fibrotic steatohepatitis in SHRSP5/Dmcr rats before and after the appearance of fibrosis.

## Materials and Methods

### Animals

All animal experiments were conducted in compliance with the Guidelines for Animal Experiments of the Kinjo Gakuin University Animal Center. The protocol was approved by the Committee on Ethics of Animal Experiments of the Kinjo Gakuin University Animal Center (approval nos. 27 and 34). Male offsprings of the SHRSP5/Dmcr rats used in this experiment were obtained by mating males and females of the strain with high serum total cholesterol (TC) levels, as previously described [21]. All the rats were housed in a temperature- and light-controlled environment (23°C ± 2°C, 55% ± 5% humidity, 12 h light/dark cycle) with free access to the control diet and tap water ad libitum.

### Diets

Control and HFC diets were obtained from Funabashi Farm (Chiba, Japan), the compositions of which were shown in a previous report [21].

### Experimental protocols

**Fig 1:**
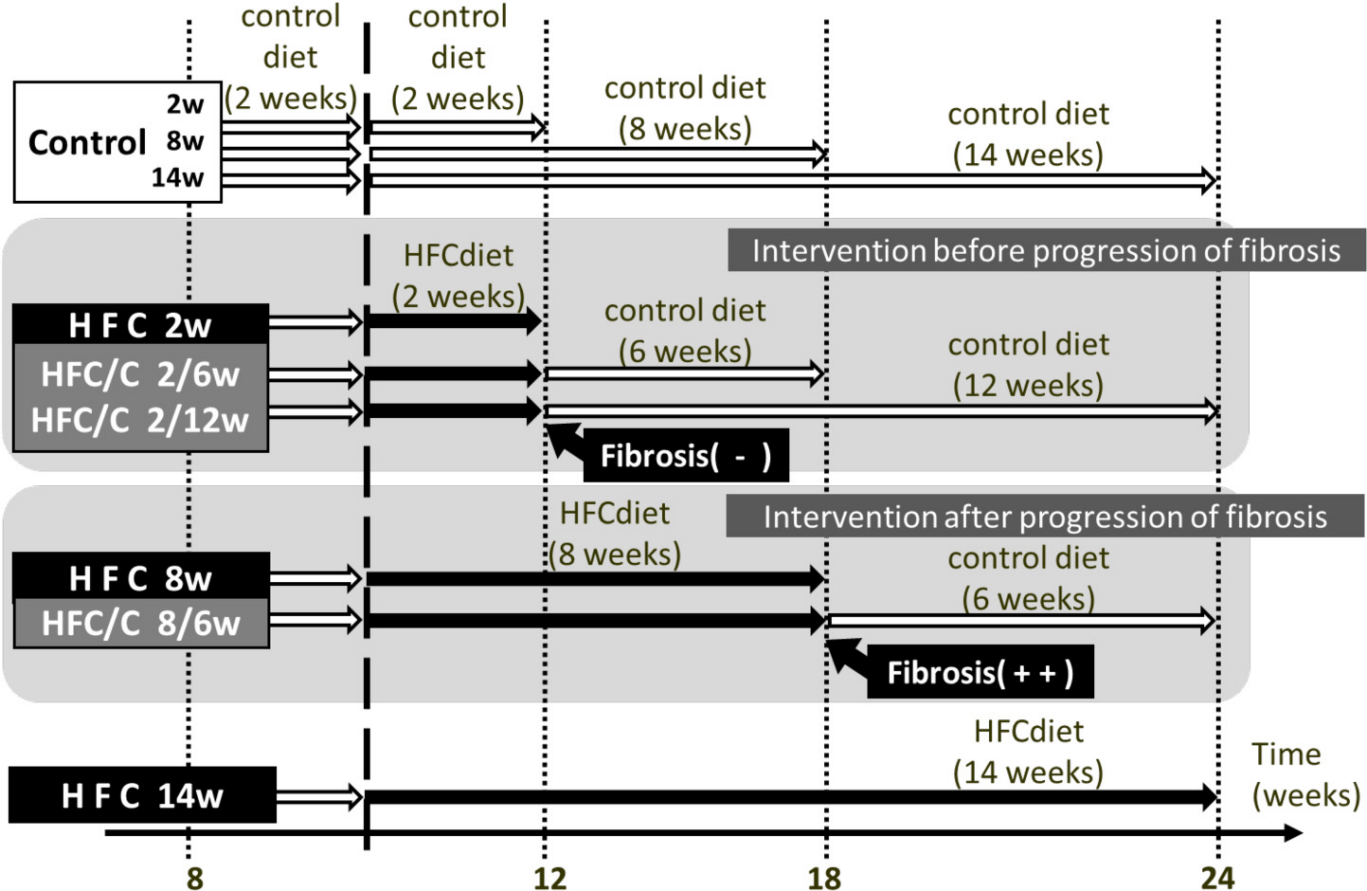
Experimental overview of control diet intervention. In this experiment, 9 groups of rats were used. Rats were fed a control diet (control group) or high-fat and -cholesterol (HFC) diet (HFC group) for 2, 8 or 14 weeks. Rats fed a control diet for 2, 2, and 8 weeks were subsequently fed a HFC diet for 6, 12, and 6 weeks, respectively, and designated as HFC/control 2/6, HFC/control 2/12, and HFC/control 8/6 groups, respectively.

At 10 weeks of age, the male offsprings were randomly divided into 9 groups of 6 rats each; of these, 3 groups were fed a control diet for 2, 8, or 14 weeks and 3 groups were fed an HFC diet for 2, 8, or 14 weeks. The remaining 3 groups represented the dietary intervention groups; these groups were fed a control diet for 6, 12, or 6 weeks after HFC diet feeding for 2, 2, or 8 weeks, and the groups were accordingly designated as the HFC/control (2/6, 2/12, or 8/6, respectively) groups (Fig 1). According to a previous report, this animal strain does not develop liver fibrosis on HFC diet feeding for 2 weeks; however, it develops severe fibrosis on feeding of 8 weeks [21]. This means that the HFC/control 2/6 and 2/12 groups underwent dietary intervention prior to the appearance of hepatic fibrosis, whereas the 8/6 group underwent dietary intervention after the progression of hepatic fibrosis. All rats had free access to their respective test diet and water. After 18–20 h of fasting from the last feeding, rats were weighed, anesthetized using pentobarbital (70 mg/kg), and sacrificed. Blood samples were collected, and the livers were removed and weighed. A portion of each liver was fixed using 10% buffered formalin for histopathological analysis, and fresh tissue was stored at −80°C until use. Serum was prepared by centrifugation of the blood samples at 3500 ×*g* for 10 min and stored at −80°C until use.

### Histopathology

The formalin-fixed liver tissue samples were subjected to hematoxylin and eosin (H&E) staining, Azan staining for representative photos, and Elastica van Gieson (EVG) staining using Sirius red stain for calculating the fibrosis area. Tissue sections were examined by light microscopy using DM750 (Leica, Wetzlar, Germany). The fibrotic area of EVG-stained sections was evaluated using NIS-Elements software (Nikon Instruments, Tokyo, Japan).

### Immunohistological staining for CD68- and α-SMA-positive cells

The formalin-fixed liver tissue samples were immunostained for CD68 and α-smooth muscle actin (SMA). Tissue sections were deparaffinized and unmasked using Histo/Zyme (Dagnostic BioSystems, Pleasanton, CA). They were subsequently washed using phosphate-buffered saline (PBS) and a solution containing H_2_O_2_ to block endogenous peroxidase activity. The sections were incubated overnight with anti-CD68 (Abcam plc, Cambridge, UK) or anti-α-SMA (Abcam plc, Cambridge, UK) antibodies; this was followed by their incubation with the secondary antibody MAX-PO (MULTI) (Nichirei Biosciences Inc., Tokyo, Japan). After washing with PBS, color was developed using peroxidase substrate ImmPACT^TM^ DAB (Vector Laboratories, Burlingame, CA) and observed by light microscopy using DM750.

### Biochemical analysis of serum and liver tissue extracts

Serum triacylglycerol (TG), TC, aspartate aminotransferase (AST), and alanine aminotransferase (ALT) levels were determined by SRL Inc. (Tokyo, Japan). Hepatic lipid was extracted as described by Folch *et al*. [29], and hepatic TG and TC contents were measured using the Triglyceride E-test wako and Cholesterol E-Test wako kits (Wako, Osaka, Japan), respectively.

### Hydroxyproline assay

Liver tissues were hydrolyzed in 6N hydrochloric acid, and the hydroxyproline contents were determined using the QuickZyme Hydroxyproline Assay (COSMO BIO, Tokyo, Japan).

### Quantitative real-time PCR

Total RNA was isolated from whole livers using the RNeasy Mini Kit (QIAGEN, Tokyo, Japan). Real-time PCR analysis was performed using 2-step RT-qPCR System (PROMEGA, Tokyo, Japan) and ABI PRISM 7000 (Applied Biosystems, Foster City, CA). We normalized the mRNA expression levels to glyceraldehyde 3-phoshate dehydrogenase (GAPDH) mRNA levels in the same preparation using the ΔΔCT method. The primer sequences are listed in the S1 Table.

### Western blot analysis

Sections of liver tissue were homogenized using 3 volumes of 0.25 M sucrose–10 mM PBS (pH 7.4). The samples were subjected to 10% sodium dodecyl sulfate-polyacrylamide gel electrophoresis, as previously described [30]. Membranes were incubated with the following antibodies: diacylglycerol acyltransferase (DGAT)-1 and -2, carnitine palmitoyltransferase (CPT)-1, acetyl-CoA carboxylase (ACC), transforming growth factor (TGF)-β1, and α-SMA. Immunoblotting using GAPDH antibodies was performed for loading controls. For the detection of specific proteins, ECL Western Blotting Detection Reagent (GE Healthcare, Buckinghamshire, UK) was used.

### Statistical analysis

Values were expressed as means ± standard deviation (SD). To evaluate the influence of HFC diet feeding, groups of rats fed HFC diet were compared to control rats of the same age using Student’s or Welch’s *t*-test. To evaluate time-dependent amelioration relative to baseline by control diet intervention, the above mentioned *t*-test or Tukey’s test was used when comparisons were made between 2 and 3 groups, respectively. The Tukey’s test were performed after ANOVA. Data with skewered distribution were normalized for further statistical analysis using logarithmic transformation. A probability (*P*) value < 0.05 was used as a criterion for statistical significance.

## Results

### Body and liver weight

HFC diet feeding induced a loss in body weight relative to the respective control groups (Table 1), as shown in a previous report [21]. The body weight of the animals in the HFC/control 2/6, 2/12, and 8/6 groups was not different from that of the animals in the control diet groups at 8, 14, and 14 weeks, respectively.

**Table 1:** Body and liver weight of rats in each group.

Feeding group	Control	HFC	Control	HFC	HFC/control	Control	HFC	HFC/control	
	2 weeks	2 weeks	8 weeks	8 weeks	2/6 weeks	14 weeks	14 weeks	2/12 weeks	8/6 weeks
Body weight (g)	263 ± 19	242 ± 13^a^	314 ± 20	269 ± 14^a^	292 ± 16	331 ± 22	279 ± 20^a^	304 ± 14	299 ± 14
Liver weight (g)	7.3 ± 0.7	11.6 ± 3.8^a^	8.3 ± 0.8	34.6 ± 3.7^a^	10.4 ± 0.7^b^	8.9 ± 0.9	38.9 ± 3.7^a^	8.9 ± 0.4^b^	17.4 ± 1.3^a^^,^^b^
Liver/body weight (%)	2.8 ± 0.1	4.8 ± 1.5^a^	2.6 ± 0.1	12.9 ± 1.2^a^	3.6 ± 0.1^b^	2.8 ± 0.1	14.0 ± 0.8^a^	2.9 ± 0.1^b^	5.8 ± 0.4^a^^,^^b^

Values are expressed as means ± SD (n = 6 rats); *P* < 0.05 was used as a criterion of statistical significance. The weight of each rat from the control diet intervention group and HFC diet feeding group was compared with the weight of each rat of the same age from the control diet feeding group.

^a^ Significantly different from the respective control group.

^b^ Significantly different from the respective HFC group.

HFC diet feeding induced liver enlargement, increasing both absolute and relative liver weights at all periods compared to control diet feeding. When 2 weeks of HFC diet feeding was replaced by 6 weeks of control diet feeding, the absolute and relative liver weights decreased to the same level as observed in by control diet feeding for 8 weeks. Additional replacement of the control diet for 6 weeks (12 weeks in total) after HFC diet feeding for 2 weeks also reset the liver weight to the same level as that observed after control diet feeding for 6 weeks. Switching to control diet feeding for 6 weeks after HFC diet feeding for 8 weeks significantly decreased the absolute and relative liver weights compared to those by HFC diet feeding for 14 weeks, but not completely.

### Lipid levels in serum and liver

Various serum and hepatic parameters in each group are shown in Table 2. HFC diet feeding for 2 weeks did not influence serum TG levels, whereas feeding for 8 weeks significantly decreased the serum TG levels. Subsequent dietary intervention for 6 weeks reset the decreased levels to those observed in the group fed the control diet for 14 weeks. HFC diet feeding significantly increased serum and hepatic TC levels in all periods. Control dietary intervention after HFC diet feeding for 2 weeks completely reset the serum TC levels regardless of the feeding duration. Intervention at 6 weeks following HFC diet feeding for 8 weeks reset the increased serum TC levels and also decreased hepatic TC levels; however, a complete reset of the increased TC levels was not observed.

**Table 2:** Various serum and hepatic parameters of rats in each group.

Feeding group	Control	HFC	HFC/control		Control	HFC	HFC/control	Control	HFC
	2 weeks	2 weeks	2/6 weeks	2/12 weeks	8 weeks	8 weeks	8/6 weeks	14 weeks	14 weeks
Serum TG (mg/dL)	36.2 ± 13.1	41.2 ± 5.4	37.2 ± 6.7	37.7 ± 13.5	37.3 ± 9.3	28.5 ± 11.1^a^	39.2 ± 18.1	41.8 ± 7.7	79.2 ± 62.7
Serum TC (mg/dL)	50.8 ± 1.6	199.9 ± 159.6^a^	59.7 ± 4.4^b^	65.7 ± 6.3^b^	61.3 ± 5.2	265.7 ± 157.1^a^	83.6 ± 10.9^b^	68.7 ± 2.8	1,593 ± 884^a^
AST (IU/L)	122 ± 12	246 ± 162^a^	89 ± 8^b^	115 ± 16^b^	105 ± 10	545 ± 208^a^	110 ± 9^b^	118 ± 21	1,121 ± 319^a^
ALT (IU/L)	47 ± 3	94 ± 14^a^	54 ± 4^b^	56 ± 15^b^	52 ± 4	220 ± 65^a^	94 ± 19^a^^,^^b^	50 ± 6	387 ± 87^a^
Hepatic TG (mg/g liver)	19.2 ± 3.0	56.3 ± 26.6^a^	41.3 ± 2.3^a^	23.7 ± 2.8^a^^,^^b^^,^^c^	18.0 ± 5.2	35.6 ± 11.6^a^	21.6 ± 3.2^b^	16.0 ± 2.8	28.3 ± 8.1^a^
Hepatic TC (mg/g liver)	2.0 ± 0.4	115.1 ± 22.6^a^	24.9 ± 7.5^a^^,^^b^	2.6 ± 0.4^a^^,^^b^^,^^c^	2.0 ± 0.1	128.8 ± 11.0^a^	62.4 ± 11.0^a^^,^^b^	1.9 ± 0.2	166.3 ± 33.3^a^

Values are expressed as means ± SD (n = 6 rats); *P* < 0.05 was considered statistically significant.

^a^ Significantly different from control group rats of the same age.

^b^ Significant differences were observed between control diet intervention for 6 or 12 weeks and HFC diet feeding for 2 or 8 weeks.

^c^ Significant differences were observed between groups of control diet intervention for 6 and 12 weeks.

TG, triacylglycerol; TC, total-cholesterol; AST, aspartate aminotransferase; ALT, alanine aminotransferase.

### Hepatic damage marker analysis

HFC diet feeding significantly elevated serum AST and ALT levels for all periods (Table 2). As previously shown, these findings suggest that HFC diet feeding induces hepatic necrosis [21,31]. Switching to the control diet for 6 weeks as well as for 12 weeks after HFC diet feeding for 2 weeks decreased AST and ALT levels to levels similar to those observed by control diet feeding. Increased serum AST levels following HFC diet feeding for 8 weeks were also reset by dietary intervention for 6 weeks. However, the dietary intervention did not completely reset the serum ALT levels to levels similar to those observed in the control group.

### mRNA levels of inflammatory cytokines

Expression of inflammatory cytokines was measured using real-time PCR system (Table 3). HFC diet increased the expression of interleukin (IL)-β1 mRNA, and peaked at 2 weeks. However after 8 weeks, the IL-β1 level had decreased to half, and no significant difference was noted at 14 weeks. Dietary intervention significantly reset the elevated IL-β1 levels at either 2 or 8 weeks into the HFC diet. The HFC diet also increased tumor necrosis factor (TNF)-α and IL-6 mRNA levels. The dietary intervention could reset the increased expression levels of both genes when applied after 2 weeks, but did not completely reset the increases after 8 weeks into the HFC diet.

**Table 3:** mRNA levels of fibrosis- and inflammation-related proteins.

Feeding group	Control	HFC	HFC/control		Control	HFC	HFC/control	Control	HFC
	2 weeks	2 weeks	2/6 weeks	2/12 weeks	8 weeks	8 weeks	8/6 weeks	14 weeks	14 weeks
IL-1β	0.7 ± 0.2	3.0 ± 1.4^a^	0.5 ± 0.1^b^	0.3 ± 0.1^b^	0.5 ± 0.2	1.1 ± 0.6	0.7 ± 0.3	0.8 ± 0.4	0.8 ± 1.5
TNF-α	0.8 ± 0.1	2.7 ± 2.0^a^	0.6 ± 0.2^b^	0.4 ± 0.1^b^	0.6 ± 0.2	1.8 ± 0.6^a^	2.2 ± 0.9^a^	0.5 ± 0.1	2.7 ± 0.8^a^
IL-6	1.1 ± 0.5	5.9 ± 6.1^a^	0.7 ± 0.4^b^	0.7 ± 0.5^b^	0.5 ± 0.4	26.7 ± 17.0^a^	10.1 ± 13.2^a^^,^^b^	0.9 ± 0.5	117.4 ± 92.2^a^
TGF-β1	1.1 ± 0.2	2.1 ± 0.2^a^	0.9 ± 0.1^b^	0.8 ± 0.1^b^	1.2 ± 0.2	4.6 ± 2.7^a^	2.8 ± 1.2^a^	1.0 ± 0.2	8.4 ± 2.3^a^
α-SMA	0.8 ± 0.3	1.0 ± 0.2	0.7 ± 0.2	0.8 ± 0.3	1.0 ± 1.0	3.4 ± 2.7^a^	0.6 ± 0.3^b^	1.0 ± 0.6	8.5 ± 4.7^a^
PDGFβR	1.0 ± 0.2	2.9 ± 0.8^a^	2.9 ± 0.7^a^	2.3 ± 0.8	1.1 ± 0.4	5.9 ± 1.4^a^	6.4 ± 3.8^a^	2.1 ± 1.2	16.6 ± 5.4^a^
Col1α1	1.0 ± 0.3	2.0 ± 1.0^a^	0.7 ± 0.3^b^	0.4 ± 0.1^b^	0.6 ± 0.2	38.3 ± 35.8^a^	9.8 ± 6.0^a^^,^^b^	0.6 ± 0.1	56.8 ± 21.9^a^
MMP-2	1.2 ± 0.2	1.2 ± 0.6	0.7 ± 0.1	0.6 ± 0.1^b^	0.8 ± 0.1	6.0 ± 3.8^a^	4.5 ± 2.6^a^	0.9 ± 0.2	13.1 ± 7.8^a^
TIMP-1	1.0 ± 0.2	2.2 ± 1.2^a^	0.6 ± 0.1^b^	0.4 ± 0.1^a^^,^^b^	1.0 ± 0.2	4.9 ± 1.7^a^	2.0 ± 0.6^a^^,^^b^	1.1 ± 0.2	9.8 ± 1.8^a^

Values are expressed as means ± SD (n = 6 rats); *P* < 0.05 was considered statistically significant. All mRNA levels were expressed relative to the glyceraldehyde 3-phoshate dehydrogenase (GAPDH) mRNA levels in the same preparation.

^a^ Significantly different from control group rats of the same age.

^b^ Significant differences were observed between control diet intervention for 6 or 12 weeks and HFC diet feeding for 2 or 8 weeks.

IL-1β and -6, interleukin-1β and -6, respectively; TNF-α, tumor necrosis factor-α; TGF-β1, transforming growth factor-β1; PDGFβR, platelet-derived growth factor receptor β; α-SMA, α-smooth muscle actin; Col1α1, α-1 type I collagen; MMP-2, matrix metallopeptidase -2; TIMP-1, tissue inhibitor of metalloproteinase -1.

### Histological findings of steatosis

**Fig 2:**
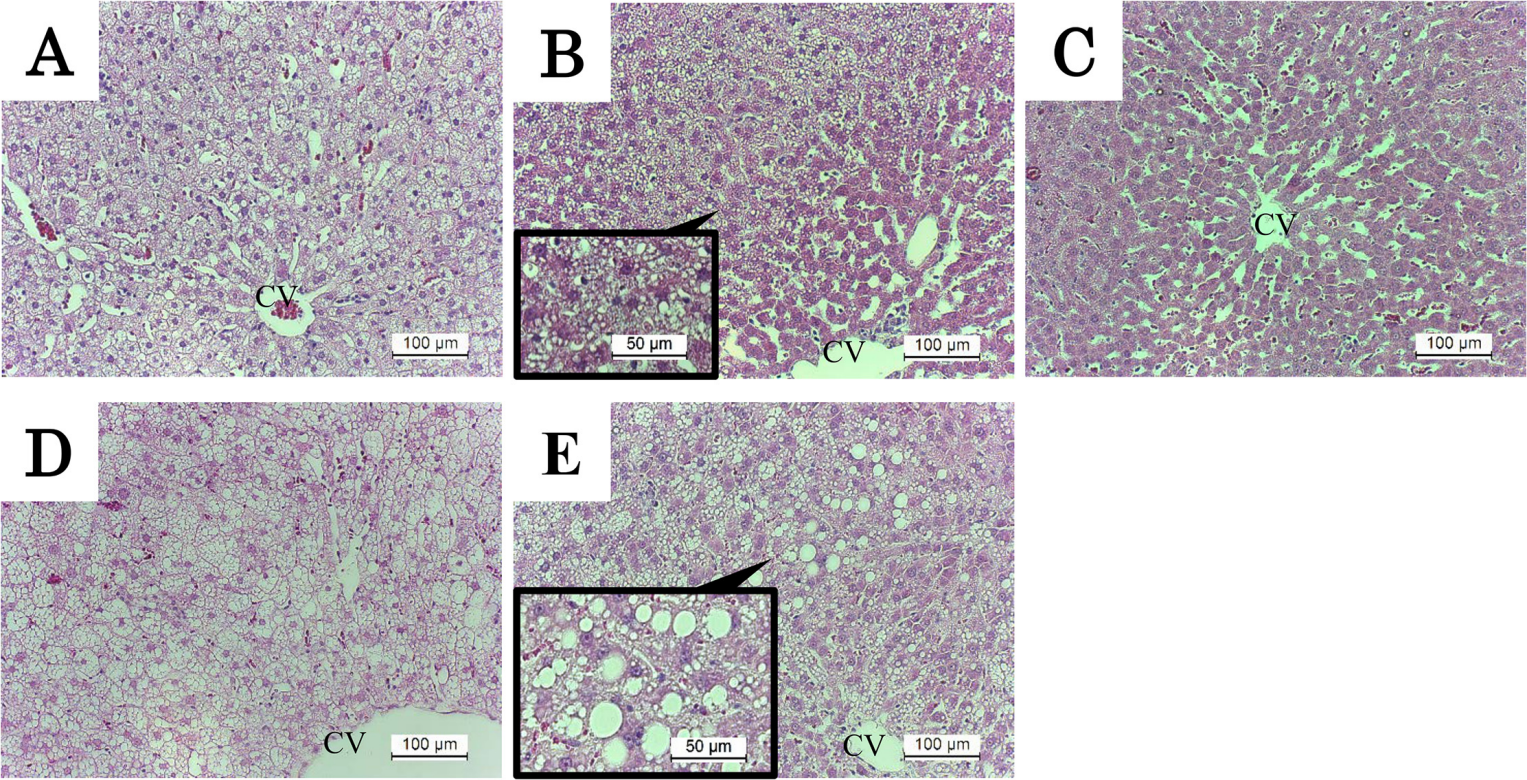
H&E staining of liver in rats. Original magnifications and expanded view were ×200 and ×400, respectively. (A) Representative image of liver tissue from rats fed HFC diet for 2 weeks, (B) and (C) control diet intervention group for 6 and 12 weeks after HFC diet feeding for 2 weeks, respectively. (D) Group fed a HFC diet for 8 weeks, (E) the control diet intervention group for 6 weeks after HFC diet feeding for 8 weeks. Lipid droplets were scattered in the liver tissue in all HFC groups (A and D). Following dietary intervention for 6 weeks after HFC diet feeding for 2 weeks, a few lipid droplets remained mainly around the central vein (expanded view in B); however, this was completely ameliorated after dietary intervention for 12 weeks (C). Dietary intervention for 6 weeks after HFC diet feeding for 8 weeks slightly ameliorated steatosis, but lipid droplets similar to macrovesicular steatosis remained in the liver (expanded view in E). CV, central vein.

HFC diet feeding markedly induced time-dependent accumulation of lipid in the liver, as shown in a previous report [21]. Switching to the control diet for 12 weeks after HFC diet feeding for 2 weeks improved steatosis, but did not completely eliminate it after an intervention period of 6 weeks (Fig 2A–2C). Switching HFC diet feeding for 8 weeks with control diet feeding for 6 weeks appeared to improve steatosis, although large lipid droplets indicating macrovesicular steatosis were observed (Fig 2D and 2E).

**Fig 3:**
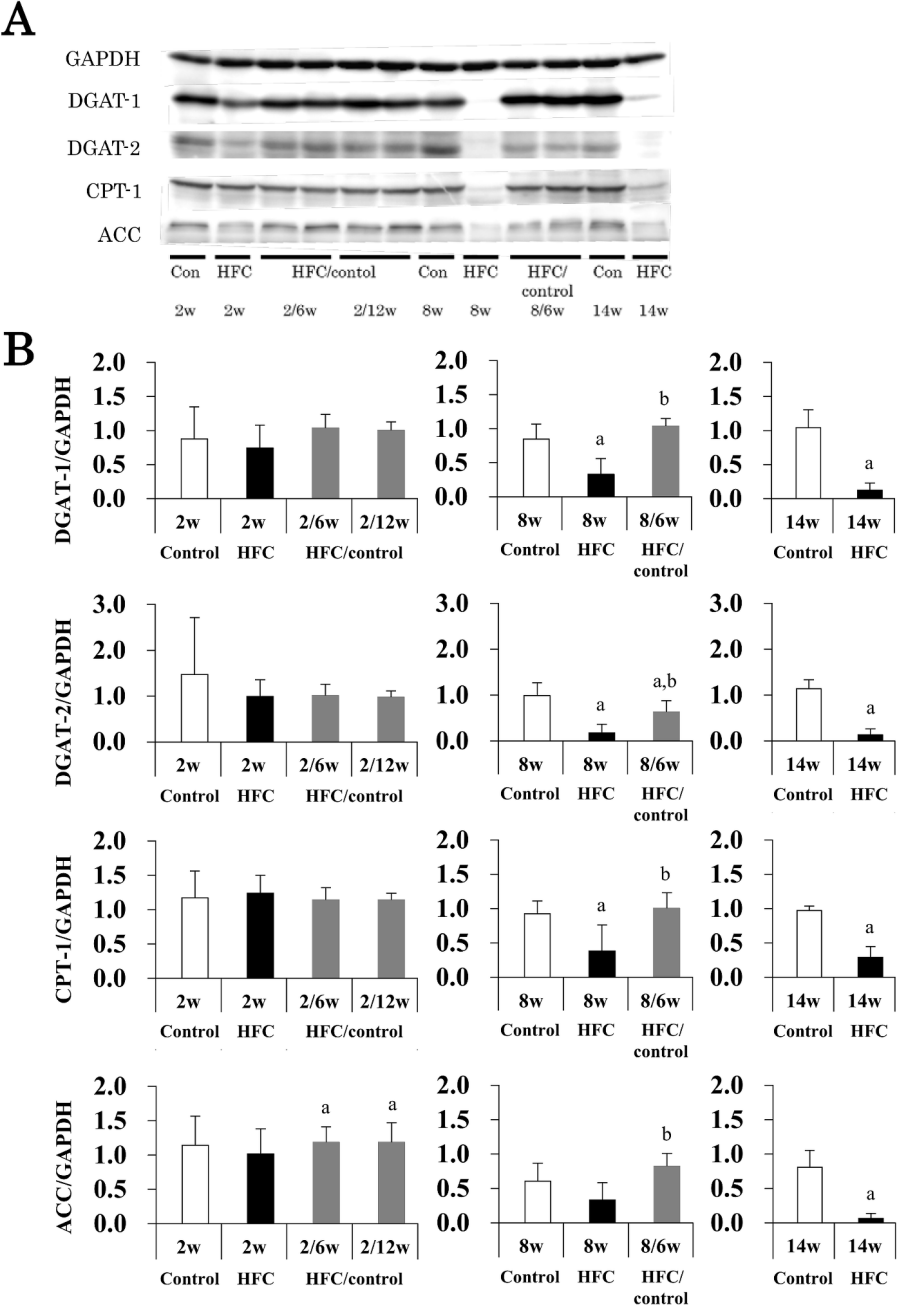
Effects of dietary intervention on lipid metabolism. Hepatic DGAT-1, DGAT-2, CPT-1, and ACC protein levels were analyzed by Western blotting (A), means and SD (n = 6) were shown by columns and bars, respectively (B). GAPDH was used as a loading control. *P* < 0.05 was used as a criterion of statistical significance. ^a^ Significantly different from the control rats of the same age. ^b^ Significant differences were observed between control diet intervention for 6 or 12 weeks and HFC diet feeding after 2 or 8 weeks.

Expression of lipid metabolism-related factors was analyzed by Western blotting (Fig 3). HFC diet feeding for 8 and 14 weeks suppressed hepatic DGAT-1 and -2 expression, which regulates TG synthesis in the hepatocytes. Switching to control diet feeding for 6 weeks after HFC diet feeding for 8 weeks improved both levels, particularly the former, which was reset to the same level as that observed in the controls. Similarly, HFC diet feeding suppressed the expression of CPT-1 and ACC, which are involved in fatty acid β-oxidation and synthesis, respectively, but control diet intervention reset expression levels to those observed in the controls. Thus, HFC diet feeding may cause lipid abnormality by inhibiting both degradation and synthesis of TG, whereas dietary intervention improved these functions and also improved steatosis.

### Influence of dietary intervention on hepatic fibrosis

**Fig 4:**
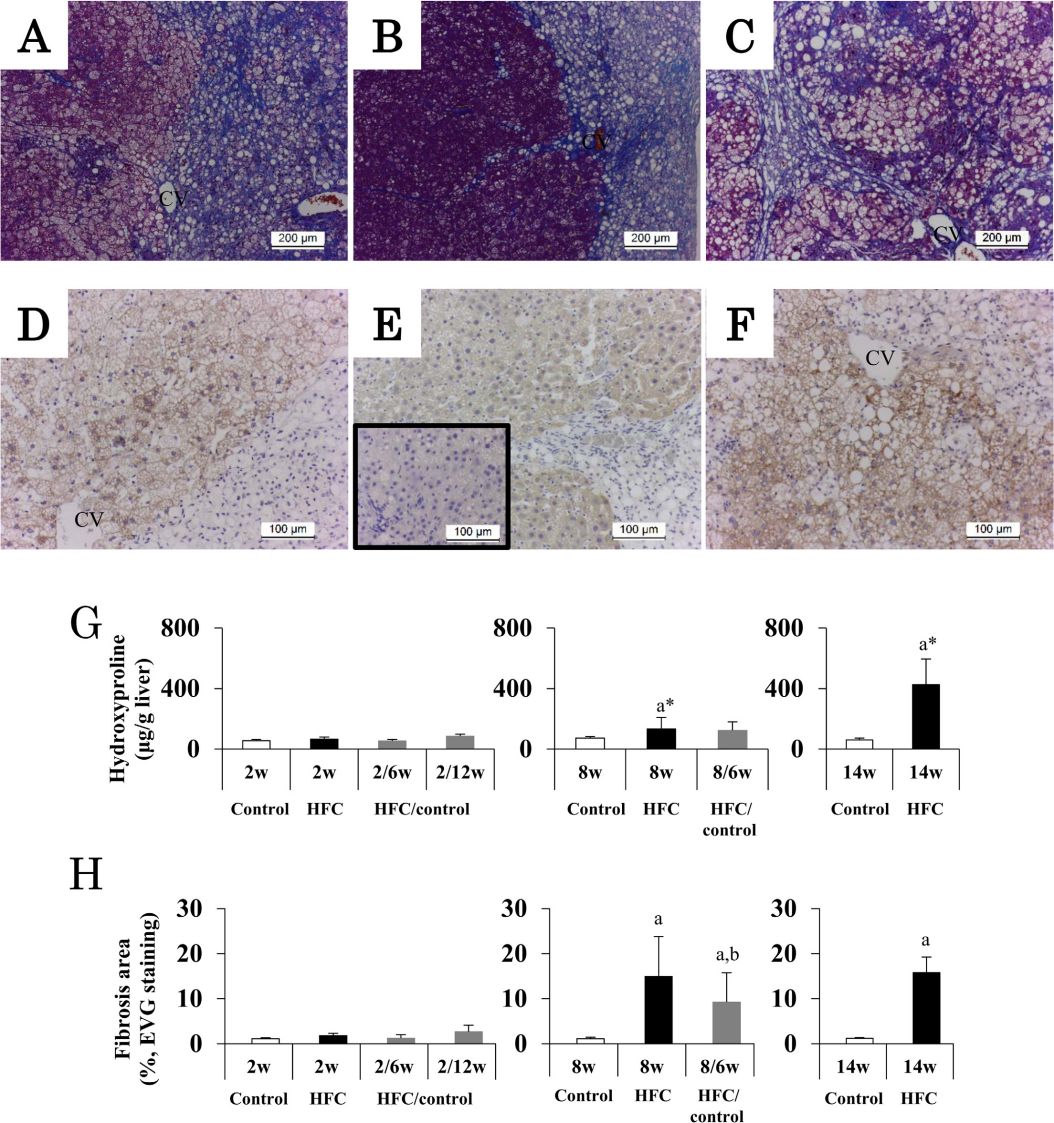
Hepatic fibrosis in HFC rats and intervention groups. Original magnifications of AZAN stain and α-SMA immunostaining were ×100 and ×200, respectively. Representative images of Azan staining, (A) liver tissue of rats fed HFC diet for 8 weeks, (B) the dietary intervention group at 6 weeks after HFC diet feeding for 8 weeks, (C) the group fed HFC diet for 14 weeks, and representative images of α-SMA immunostaining, (D) liver tissue of rats fed HFC diet for 8 weeks, (E) the dietary intervention group at 6 weeks after HFC diet feeding for 8 weeks, (F) the group fed HFC diet for 14 weeks. Small window of (E) shows the image of central region of liver. (G) and (H) show means and SD (n = 6) of hydroxyproline content and quantitative analysis of fibrotic areas using EVG staining, respectively. *P* < 0.05 was used as a criterion of statistical significance. ^a^ Significantly different from control rats of the same age. ^b^ Significantly different from HFC diet feeding for 14 weeks. * The data were analyzed using Mann-Whitney U test due to the significant variance.

Similar to a previous report [21], HFC diet feeding for 2 weeks did not induce hepatic fibrosis. HFC diet feeding for 8 weeks apparently induced severe fibrosis in the peripheral parts of liver, scattered in the right side as shown in Fig 4A. Subsequent dietary intervention for 6 weeks appeared to partially suppress the fibrosis, but the fibrosis was compressed into strips going out from the central vein (CV) area (Fig 4B). Hepatocytes in the left side of Fig 4B were stained dark because of a reduction in lipid droplets in the hepatic parenchyma. When HFC diet feeding continued for 14 weeks, the fibrosis progressed with pseudolobules (Fig 4C) and; this fibrosis appeared to be more severe than that observed in the 6-week intervention group after 8 weeks of HFC diet feeding.

HFC diet for 8 and 14 weeks increased the number of hepatic α-SMA positive cells in the area without fibrosis (Fig 4D and 4F, particularly on the left side of Fig 4D) where hepatocytes survived. These findings were also observed in the dietary intervention group (Fig 4E); however, fewer α-SMA positive cells were observed in the central area of the liver, a region far from the fibrotic area (small window in Fig 4E). HFC diet for 8 and 14 weeks slightly and markedly increased the hydroxyproline content of the liver, respectively (Fig 4G).

The diet also significantly increased the fibrosis area at 8 and 14 weeks (Fig 4H). The mean value of the fibrosis area in the group fed HFC diet for 14 weeks was not different from that of the group fed HFC diet for 8 weeks. However, the dietary intervention significantly reduced the hydroxyproline level (*P* = 0.0003) and the mean area of fibrosis (*P* = 0.0422) compared with HFC diet feeding for 14 weeks.

The mRNA expression of fibrogenesis-related genes is also shown in Table 3. The HFC diet duration dependently increased liver expression of all the studied fibrogenesis-related genes, i.e., TGF-β1, α-SMA, and platelet-derived growth factor receptor β (PDGFβR, which are involved in not only fibrogenesis but also in angiogenesis and tumorigenesis [32]); α-1 type I collagen (Col1α1) and matrix metallopeptidase -2 (MMP-2), which are involved in the degradation of collagen fiber [33]; and tissue inhibitor of metalloproteinase -1 (TIMP-1), which is an inhibitor of MMPs [33]. The dietary intervention for 6 and 12 weeks after HFC diet for 2 weeks completely ameliorated the increased expression of fibrogenesis-related genes, except PDGFβR. However, when the intervention occurred 6 weeks after fibrosis appeared, it did not normalize the expression levels, with the exception of α-SMA. It is noted that the dietary intervention after fibrosis appeared was too difficult to improve the increased level of PDGFβR and MMP-2 mRNAs at HFC diet feeding for 8 weeks.

**Fig 5:**
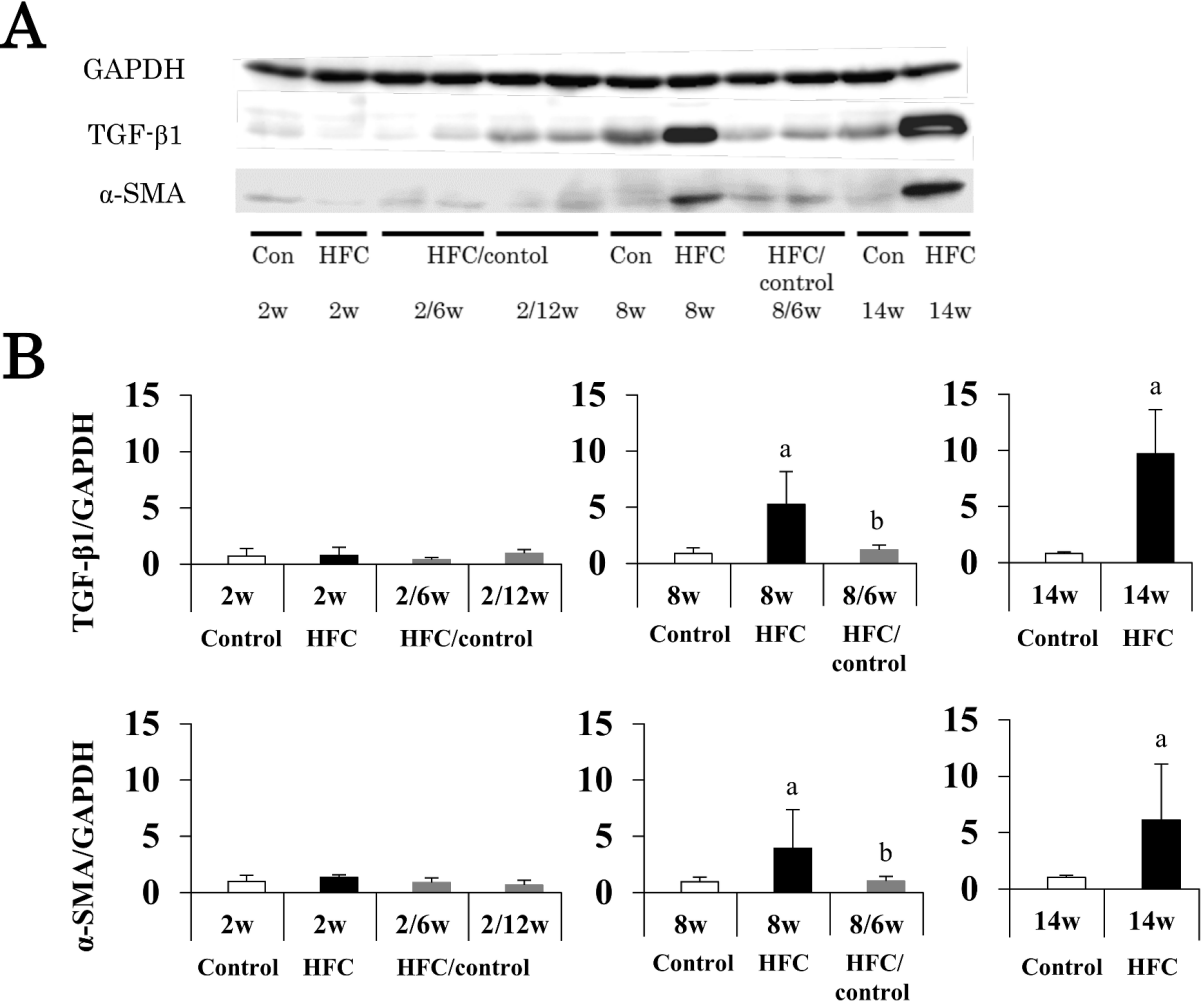
Effects of dietary intervention on fibrogenesis factors. (A) Representative Western blot results of hepatic TGF-β1 and α-SMA protein levels, and means and SD (n = 6) shown as columns and bars, respectively (B). GAPDH was used as a loading control. *P* < 0.05 was used as a criterion of statistical significance. ^a^ Significantly different from control rats of the same age. ^b^ Significant differences were observed between control diet intervention for 6 or 12 weeks and HFC diet feeding for 2 or 8 weeks.

Western blot analysis showed that protein expressions of TGF-β1 and α-SMA were elevated by HFC diet feeding for 8 weeks. Switching to control diet feeding for 6 weeks reduced protein expressions to the same levels seen during control diet feeding for 14 weeks (Fig 5).

### CD68-positive cells

**Fig 6:**
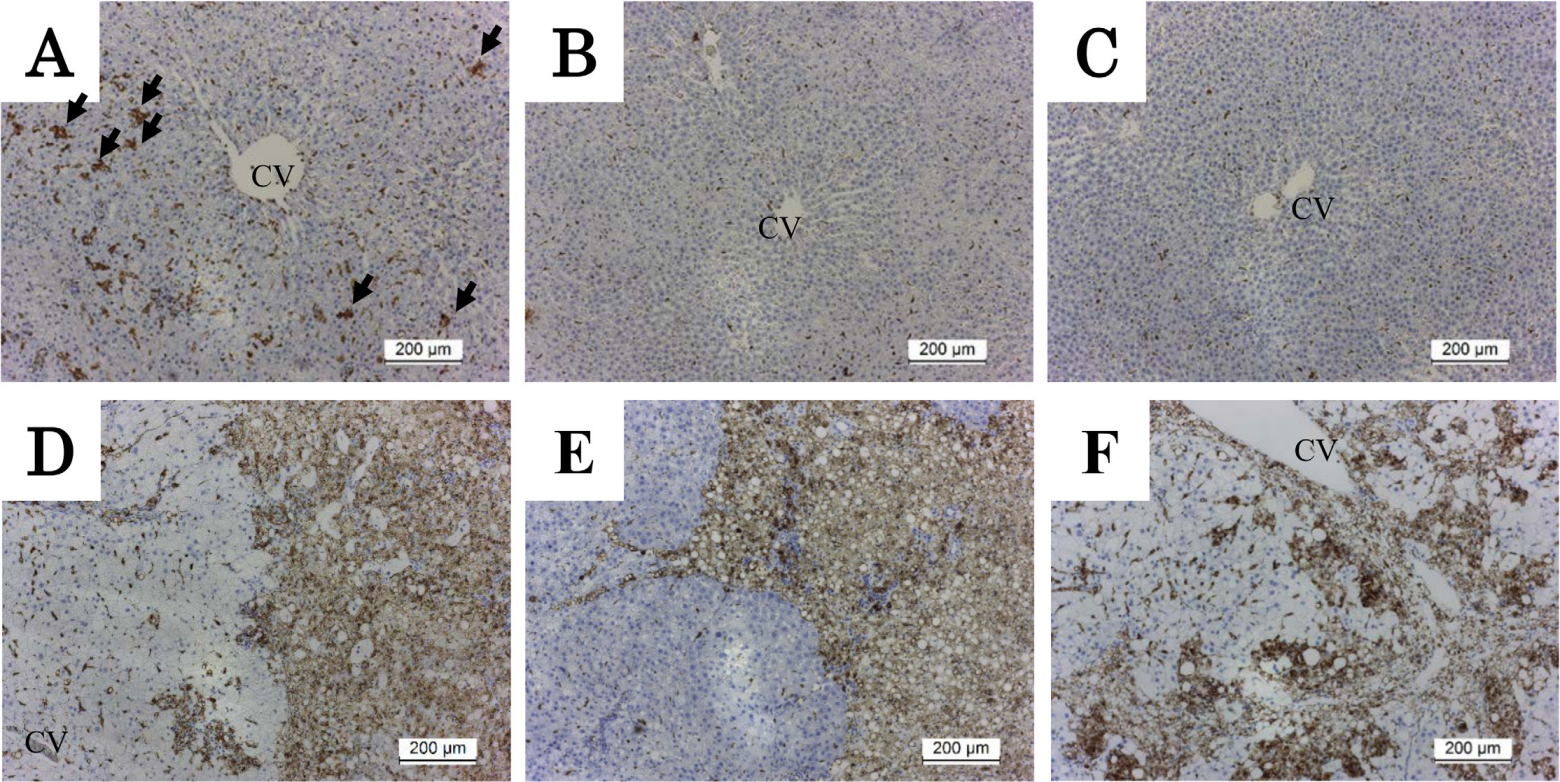
CD68 immunostaining of liver tissue in HFC rats and intervention groups. Original magnifications were ×100. (A) Representative image of liver tissue from rats fed HFC diet for 2 weeks, and (B and C) control diet intervention group at 6 and 12 weeks after HFC diet feeding for 2 weeks, respectively. (D) The group fed HFC diet for 8 weeks, (E) the intervention group at 6 weeks after HFC diet feeding for 8 weeks, and (F) the group fed HFC diet for 14 weeks. CD68-positive cells increased in the hepatic parenchyma in the HFC diet-fed group in a time-dependent manner (A, D, F). Arrows show prominent aggregation of CD68-positive cells. Subsequently, dietary intervention decreased cell aggregation compared with baseline (B, C) in a time-dependent manner, but CD68-positive cells remained around the fibrotic area in the liver even over a 6-week intervention period after appearance of fibrosis (E). CV, central vein.

To investigate the effect of dietary intervention on hepatic macrophages in the damaged liver induced by HFC diet feeding, staining for CD68-positive cells was performed. A few CD68-positive cells were observed in the liver of rats fed the control diet. HFC diet feeding for 2 weeks prominently increased aggregation of CD68-positive cells. (Fig 6A). Although subsequent dietary intervention for 6 (Fig 6B) or 12 (Fig 6C) weeks completely eliminated these cells, HFC diet feeding for 8 weeks increased the aggregation of cells more prominently in the entire hepatic parenchyma (Fig 6D). Control diet intervention for 6 weeks compressed CD68-positive cells into strips going out from the CV area (Fig 6E), which took place in a manner considerably similar to fibrosis development (Fig 4B). HFC diet feeding for 14 weeks apparently increased the number of CD68-positive cells in the entire area of the liver (Fig 6F).

## Discussion

It has been difficult to test the efficacy of dietary intervention for improving diet-induced steatohepatitis with fibrosis using an animal model because no relevant animal model has been available to monitor changes from hepatic steatosis to hepatitis and fibrosis by diet feeding only. We have recently established a diet-induced fibrotic steatohepatitis SHRSP5/Dmcr model, the so-called NASH model [21]. Using this model, we found that when control diet intervention took place at the hepatic steatosis and hepatitis stage, but prior to the appearance of fibrosis (HFC diet feeding for 2 weeks), the intervention enabled recovery of the liver except for the increased hepatic TG and TC levels and PDGFβR expression. However, when control diet intervention was introduced only at the stage of the appearance of fibrosis, i.e., after HFC diet feeding for 8 weeks, the intervention could neither completely reset serum ALT and hepatic TC levels and fibrosis nor the elevated expression of many inflammatory- and fibrosis-related genes such as TNF-α, IL-6, TGF-β1, PDGFβR, Col1α1, MMP-2, and TIMP-1. However, the intervention reset the elevated serum TG, TC, and AST levels as well as the hepatic TG and α-SMA levels. Thus, the present study suggests that dietary intervention is useful because it completely or partially improved several serum and hepatic inflammatory- and fibrosis-related parameters that indicate HFC diet-induced hepatic damage.

Several studies have demonstrated that lifestyle intervention successfully improved steatohepatitis. Ueno *et al*. showed that dietary energy control and exercise therapy for 3 months improved AST, ALT, and histological steatosis [23], and Haung *et al*. demonstrated that one year of lifestyle counseling improved steatosis and hepatitis, although some hepatic fibrosis remained [34]. Likewise, dietary and physical intervention for NASH patients improved liver histological findings except fibrosis [24]. Taken together, although well-controlled nutritional intervention is considered to be one of the most effective treatments for steatohepatitis, in patients with already developed steatohepatitis and fibrosis, additional medication may be required.

Because HFC diet contains a high level of cholesterol [21], human NASH patients were also found to have a much higher dietary cholesterol consumption compared with healthy individuals [35]. Furthermore, cholesterol consumption is significantly associated with a risk of developing cirrhosis or liver cancer [36]. In an animal study, cholesterol accumulation was considered to be susceptible to stimulation of TNF-α as opposed to TG accumulation [37]. In another study, additional cholesterol added to chow induced NASH with hepatocyte ballooning and hepatic fibrosis, although high-fat diet feeding led only to simple steatosis [38]. This means that cholesterol consumption is important in the pathogenesis and progression of NAFLD/NASH. In the present study, dietary intervention after the appearance of hepatic fibrosis could not reset hepatic TC levels to the control level, and the TC may have remained as droplets as in macrovesicular steatosis. Nonobese human NAFLD patients consume much more dietary cholesterol than obese patients [39]. Taken together, control of dietary cholesterol consumption along with energy control is important, particularly in the treatment of nonobese NAFLD/NASH patients.

In the present study, hepatic fibrosis persisted despite intervention with the control diet. TGF-β1 is a stimulus for extracellular matrix production by stellate cells, the activation of which is indicated by α-SMA expression [40], and Col1α1 is a common hallmark of fibrosis [41]. Dietary intervention suppresses the progression of fibrosis because it resets the expression of both TGF-β1 and α-SMA, although it did not reset the Col1α1 mRNA levels. The intervention slightly reduced the fibrotic area than that observed in rats continuously fed HFC diet, wherein bridged fibrosis was observed. Although the fibrotic area was compared with that of baseline rats having undergone 8-week HFC diet feeding, the intervention did not make any significant difference. MMP-2 is an enzyme that degrades extracellular collagen, and expression of its inhibitory factor, TIMP-1, was increased by HFC diet. The former was unaffected, whereas the latter was decreased to half after the dietary intervention. Therefore, fibrosis degradation may predominate when the dietary intervention is applied for 6 weeks after the appearance of fibrosis, although the degradation observed was minimal. Similar to the MMP-2 results, the 6-week dietary intervention did not ameliorate the increased expression of PDGFβR, regardless of the periods of HFC diet feeding. Okada *et al*. reported that peretinoin administration inhibited levels of this growth factor and reduced fibrosis [42]. To heal the formed fibrosis completely, longer intervention periods with a control diet or a control diet together with some medication such as peretinoin may be needed. However, it was noted that the scattered fibrosis was accumulated in zones going out from the CV area after control diet intervention in the present study.

We also performed immunostaining of CD68-positive cells to determine the macrophages’ function in the SHRSP5/Dmcr model. CD68 is specific marker of Kupffer cells, and its activation plays an important role in hepatic inflammation and fibrosis [43]. Moreover, CD68-positive cells have been reported to be correlated with pathologic severity in patients with NAFLD [44]. In the present study, prominent and aggregated CD68-positive cells were already observed throughout the hepatic lobules of rats fed HFC diet for 2 weeks, although no prominent appearance of fibrosis was observed. The dietary intervention at this point completely eliminated these aggregated cells, whereas alternatively induced elongated CD68-positive cells along the lines of the sinusoidal area. Lefkowitch *et al*. reported that spindle-shaped CD68-positive cells were observed in normal liver and simple steatosis [45], suggesting that intervention before the appearance of fibrosis improved the inflammatory reaction related to macrophages to the level observed in a normal liver and simple steatosis. This finding was very similar to changes observed in inflammatory cytokine genes. On the other hand, dietary intervention for 6 weeks after the appearance of fibrosis could not completely remove the aggregated CD68-positive cells, which were almost observed only in an area corresponding to that of the fibrosis in the hepatic lobules. This means that the intervention also had a beneficial effect on HFC-induced inflammatory reaction in the hepatocytes even after the appearance of the fibrosis, whereas it could not completely remove the aggregated CD68-positive cells around the fibrotic area. This may be related to the finding that after fibrosis the intervention appeared not to completely reset expression of the inflammatory cytokines TNF-α and IL-6 in the liver. Although serum AST and ALT values are generally used as biomarkers for hepatitis, these values have been reported not to correlate with the severity of hepatic fibrosis [44,46,47]. AST levels were found neither related to the active macrophage status nor fibrosis in the present study because the levels were completely reset to control levels by the dietary intervention both before and after the appearance of fibrosis. Therefore, consecutive treatment may be required even if AST levels return to normal levels. Itagaki *et al*. reported that methionine-choline-deficient-diet feeding for 16 weeks induced hepatic fibrosis and aggregated CD68-positive cells, and these findings were not reset even after control dietary intervention lasting for 2 weeks [47]. Similar to the findings of the present study, they described that the presence of fibrosis and aggregated CD68-positive cells, using SHRSP5/Dmcr rats and HFC diet, showed signs of irreversible late-stage steatohepatitis. However, in contrast to the report by Itagaki *et al*., we also observed the aggregated CD68-positive cells in the liver before the appearance of hepatic fibrosis. Therefore, we speculated that the aggregated cells may not always be related to the appearance of hepatic fibrosis. Until recently, hepatic fibrosis has been considered an irreversible degeneration; however, nowadays, evidence of its reversibility is being reported [48]. Further studies are needed to solve the relationship between hepatic fibrosis and potentially associated function(s) of macrophages.

In conclusion, dietary intervention is effective for improving dietary fibrotic steatohepatitis. In the present animal model, intervention was fully effective for liver steatosis and hepatitis without fibrosis. Even in fibrotic hepatitis, the intervention was partially effective on fibrosis, altering gene expression, presence of CD68-positive cells, inflammatory cytokines, and serum ALT and hepatic TC levels. Therefore, controlled diet intervention may well be the first choice of treatment for NASH/NAFLD.

## Supporting Information

S1 Fig EVG staining of liver in rats of HFC and HFC/control group. Original magnifications were ×100. (A) and (B) Representative image of liver tissue from rats fed HFC diet for 8 and 14 weeks, respectively, (C) control diet intervention group for 6 weeks after HFC diet feeding for 8 weeks.
(PDF)

S1 Table List of primers used for real-time quantitative PCR. GAPDH, glyceraldehyde 3-phoshate dehydrogenase; IL-1β and -6, interleukin-1β and -6; TNF-α, tumor necrosis factor-α; TGF-β1, transforming growth factor-β1; PDGFβR, platelet-derived growth factor receptor β; α-SMA, α-smooth muscle actin; Col1α1, α-1 type I collagen; MMP-2, matrix metallopeptidase -2; TIMP-1, tissue inhibitor of metalloproteinase -1.
(DOCX)
