# Supplementary figures and images for: Efficacy of Dietary Lipid Control in Healing High-Fat and High-Cholesterol Diet-Induced Fibrotic Steatohepatitis in Rats
Source: PLoS One. 2016 Jan 4;11(1):e0145939. doi: 10.1371/journal.pone.0145939 (PMC4699821; doi:10.1371/journal.pone.0145939)

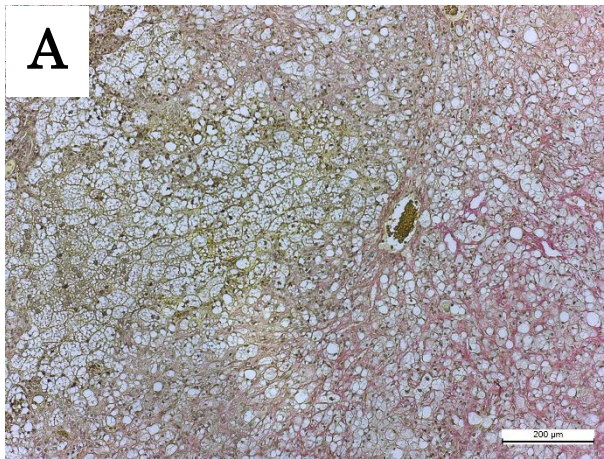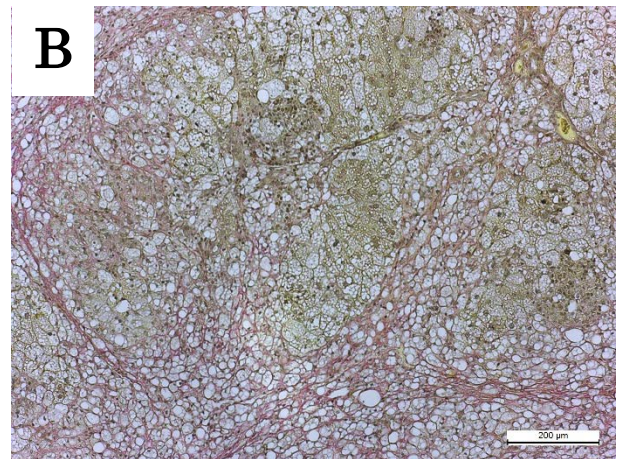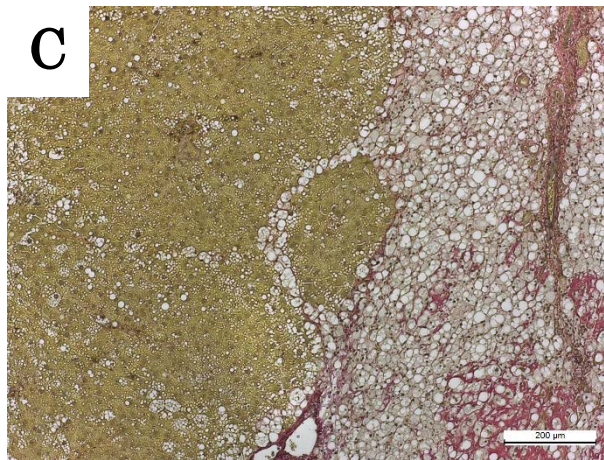

**S1 Fig. EVG staining of liver in rats of HFC and HFC/control group**

Supplement: S1 Fig — Original magnifications were ×100. (A) and (B) Representative image of liver tissue from rats fed HFC diet for 8 and 14 weeks, respectively, (C) control diet intervention group for 6 weeks after HFC diet feeding for 8 weeks. (PDF) [file pone.0145939.s001.pdf]
