# Supplementary material for: Efficacy of Dietary Lipid Control in Healing High-Fat and High-Cholesterol Diet-Induced Fibrotic Steatohepatitis in Rats
Source: PLoS One. 2016 Jan 4;11(1):e0145939. doi: 10.1371/journal.pone.0145939 (PMC4699821; doi:10.1371/journal.pone.0145939)
Supplement: S1 Table — GAPDH, glyceraldehyde 3-phoshate dehydrogenase; IL-1β and -6, interleukin-1β and -6; TNF-α, tumor necrosis factor-α; TGF-β1, transforming growth factor-β1; PDGFβR, platelet-derived growth factor receptor β; α-SMA, α-smooth muscle actin; Col1α1, α-1 type I collagen; MMP-2, matrix metallopeptidase -2; TIMP-1, tissue inhibitor of metalloproteinase -1. (DOCX) [file pone.0145939.s002.docx]

**S1 Table. List of primers used for real-time quantitative PCR**

| Genes | Forward (5’–3’) | Reverse (5’–3’) |
| --- | --- | --- |
| GAPDH | AGAACATCATCCCTGCATCCA | CCGTTCAGCTCTGGGATGAC |
| IL-1β | GAAACAGCAATGGTCGGGAC | TCAGAGGCAGGGAGGGAAA |
| TNF-α | GACCCTCACACTCAGATCATCTTCT | TGCTTGGTGGTTTGCTACGA |
| IL-6 | CAAGTCCGGAGAGGAGACTTCA | ACAATCAGAATTGCCATTGCACA |
| TGF-β1 | CAACAATTCCTGGCGTTACCTT | GACGTCAAAAGACAGCCACTCA |
| α−SMA | ATGGGCCAAAAGGACAGCTA | TGATGATGCCGTGTTCTATCG |
| PDGFβR | GCACCGAAACAAACACACCTT | ATGTAACCACCGTCGCTCTC |
| Col1α1 | ATGCTTGATCTGTATCTGCCACAAT | ACTCGCCCTCCCGTTTTT |
| MMP-2 | TGAGCTCCCGGAAAAGATTG | CATTCCCTGCGAAGAACACA |
| TIMP-1 | TACCAGAGCGATCACTTTGCCT | GAGACCCCAAGGTATTGCCAG |
